# Congenital malformations in brachycephalic dogs: A retrospective study

**DOI:** 10.3389/fvets.2022.981923

**Published:** 2022-10-04

**Authors:** Marina Vilela Estevam, Samara Beretta, Nathalia F. Smargiassi, Maricy Apparício, Gilson Helio Toniollo, Gener T. Pereira

**Affiliations:** ^1^Service of Obstetrics and Animal Reproduction (SORA), São Paulo State University—FCAV Unesp, Jaboticabal, SP, Brazil; ^2^Department of Gynecology and Obstetrics, University of São Paulo—FMRP USP, Ribeirão Preto, SP, Brazil; ^3^Department of Exact Sciences, São Paulo State University—FCAV Unesp, Jaboticabal, SP, Brazil

**Keywords:** neonate, congenital defects, inbreeding, anasarca, palatoschisis

## Abstract

The popularity of brachycephalic dogs has increased in recent years due to their docile temperament and peculiar features. The historical inbreeding and consequent lack of genetic diversity involved in the development of these breeds led to an increase in the manifestation of deleterious genes that may lead to malformations. In addition, there are serious health issues intrinsic to the conformation, mainly attributed to these extreme characteristics. Therefore, this retrospective study aimed to observe the frequency of malformations in brachycephalic dogs compared to the pure and mixed breeds (MB). The medical records of pregnant bitches admitted at the Service of Obstetrics and Animal Reproduction (SORA) from January 2017 to December 2021 were retrieved from the hospital's computer system and analyzed one by one. Seven hundred sixty-eight neonates born from 168 litters were included in this study. Of these litters, 72.6% (122/168) were brachycephalic. Malformations were found in 52 puppies, with an incidence of 6.77% (52/768). Of the 32 litters that produced malformed puppies, 28 were brachycephalic (87.5%). In total, 23 types of malformations were registered, the most common being cleft palate (1.30%) and anasarca (1.17%). Ten of the puppies (10/52; 19.23%) presented two or more associated malformations. Bitches above 7 years were more prone to present malformed puppies in their litters. Brachycephalic breeds were 3.03 times more likely to present malformed neonates when compared to other breeds; the odds ratio increased to 5.07 when modern brachycephalic was compared to ancestral brachycephalic. Regarding the mode of delivery, elective cesarean sections accounted for 66.6% of births while 19.64% were eutocic vaginal deliveries, and 13.69% were dystocic. The presence of malformed puppies in a litter causes suffering for the owner, the bitch and for the puppy itself, therefore, the veterinarian plays a key role in this scenario. Knowledge about congenital abnormalities, their causes, diagnosis, and approach is essential to reduce the incidence of malformations and improve the quality of life of these animals.

## Introduction

A congenital defect is a deviation from normal morphology or function that occurs during pregnancy and is severe enough to interfere with viability or the physical well-being of the offspring and represents one of the main causes of neonatal mortality (1, 2). A study conducted in Australia suggests that the incidence of congenital defects is about 2.8% and the mortality among malformed puppies can reach 15% (1). In Brazil, a study described 27 types of malformations in 23 dog breeds, in which 24.7% of the litters presented some type of malformation, and the incidence among the neonates was 6.7% (3). The most common malformation described was cleft palate (2.8%) and hydrocephalus (1.5%) (3).

The causes may be genetic, iatrogenic, nutritional, or infectious (2, 4). Griseofulvin, corticosteroids, antibiotics, and even aspirin have been listed as teratogenic, as well as hypervitaminosis A and D (2, 4). Some authors point out the influence of geographic regions, which directly impact exposure to pathogens, as well as differences in nutritional management and different genetic lines (4). Indeed, geographic regions can be an important factor, once infectious diseases related to malformations can be endemic in certain areas and very rare in others. Differences in breeding methods, such as the use of supplements and nutritional support, can also compose the geographic factor since the availability of ingredients varies between regions. The geographical issue also includes different genetic lineages: micro populations of the same breed are genetically isolated from each other reducing genetic variability.

There are three critical periods in fetal development (5): the first one is preimplantation (Days 2–17), in which serious injuries may result in embryonic loss. The second is when organogenesis occurs (Days 19–35) and is an important period when birth defects develop. The third is the fetal period (Days 35 to birth), during which the growth and maturation of organ systems occur. Gross structural defects are uncommon in this phase, except in structures undergoing rapid growth and maturation such as the palate, the cerebellum, and parts of the cardiovascular and urogenital system (2).

Recent studies report a higher incidence of orofacial and vertebral malformations in purebreds, especially in brachycephalics such as English and French Bulldogs, Pugs, and Boston Terriers (2, 4, 6, 7).

Although we have observed an increase in the notification of these cases in recent years (8–10) and many cases in clinical routine, there is no data on the real incidence of malformations and no studies comparing the incidence in brachycephalic dogs with other breeds.

The objective of this retrospective study was to describe the most common congenital anomalies found in litters born in our veterinary hospital from January 2017 to December 2021 and to determine if there is an association between the incidence of neonatal malformations and brachycephalic breeds.

## Materials and methods

### Data collection and analysis

All medical records of patients admitted to the veterinary hospital “Governador Laudo Natel” have been contained in a computerized system since 2017. Pregnant patients are attended exclusively at SORA (Service of Obstetrics and Animal Reproduction) regardless of other concomitant conditions. Patients admitted for other reasons and later diagnosed as pregnant are immediately referred to our service. To carry out this study, all medical records of pregnant bitches were analyzed one by one. Only the medical records of females who underwent pre-natal care and delivery/cesarean section from January 2017 to December 2021 were included in the statistics.

Data such as breed, age, breeder, and previous medical history were obtained from the records contained in the system. Data obtained from physical, laboratory, and imaging examinations, mode of delivery, and the presence of malformed fetuses were recorded. Other complementary information obtained from the zootechnical records of the breeder was also collected.

The dams included in this study were primiparous and multiparous, from different breeds, and were between 6 months and 9 years old.

The data obtained from the bitches' medical records were cataloged according to the breed and age, mode of delivery, number of puppies, number and type of malformations, and previous history. Although each animal has a medical record with the data on all attendances, the information collected was tabulated in a way that each row corresponded to a different litter, so if one bitch was attended in two different pregnancies, each birth and litter was counted separately. Twenty-nine medical records of females who underwent cesarean section did not contain enough information so could not be included. Puppies that were not born at the hospital, but were later brought in for care due to malformations were also excluded from this study.

### Pre-natal care, delivery, and neonatal care

Pre-natal care at SORA encompasses physical examination (heart rate, respiratory rate, blood pressure, body temperature, and blood glucose), laboratory tests (blood count and serum biochemistry—ALT, creatinine, total protein, and albumin), ultrasounds performed on days 30, 45, and 55 of pregnancy, and abdominal radiography for the fetal count on day 55. Additional tests were made when necessary.

The litters considered in this study were followed up and born in SORA by vaginal delivery or c-section. Neonates were evaluated at birth by Apgar score (11) and inspected for the presence of malformation and those with alterations were included in the statistical analysis. The diagnosis of malformations was made by inspection in most cases, but also using other methods such as laboratory and imaging (Ultrasound, radiography, and echocardiography) when necessary. Malformed puppies amenable to interventions were monitored and treated by the SORA staff and those with conditions incompatible with life were euthanized. Puppies that died were sent for necropsy whenever possible.

### Statistical analysis

First, the data obtained from the medical records were placed in an Excel table [Microsoft Office Excel 2019 (16.0); Microsoft Corp.] and a descriptive statistic was performed, in which the frequency of attendance between pure breeds and mixed breeds was observed, in addition to the frequency and types of malformations among the breeds. The mode of delivery were also analyzed concerning breed conformation and the presence of malformations.

In the descriptive statistics, we noticed a high frequency of malformations among brachycephalic breeds, especially in the modern ones, much higher than that observed in other purebred dogs. For this reason, in a second moment, the odds ratio was calculated comparing brachycephalic to other breeds and extreme brachycephalic to ancestral brachycephalic breeds.

The odds ratio, as well as the confidence interval, were calculated using the R program (R i386 4.1.3) Fisher's test was performed to test the association between the analyzed variables. Values were considered as significant at *p* < 0.05.

## Results

A total of 168 medical records of parturition/c-section were eligible for enrollment, and 152 corresponded to purebred litters (21 AKC recognized breeds) and 16 to mixed breeds. Brachycephalic breeds accounted for 72.6% (122/168) of the litters.

Congenital anomaly (CA) was present in 19.04% (32/168) of the litters and 87.5% of these (28/32) were brachycephalic. Twenty-one litters (21/32; 65.6%) with malformed neonates corresponded to commercial breeders and two of the dams already had a previous history of malformed puppies. Two mixed-breed neonates presented multiple congenital malformations: anencephaly, palatoschisis, eyelid aplasia, and macroglossia; the dam was a Lhasa apso accidentally mated to an American Pit Bull. Seventeen litters had stillborn with no apparent malformations. Unfortunately, not all of them could be referred for necropsy, and those that were did not show any macroscopic alterations.

**Figure 1:**
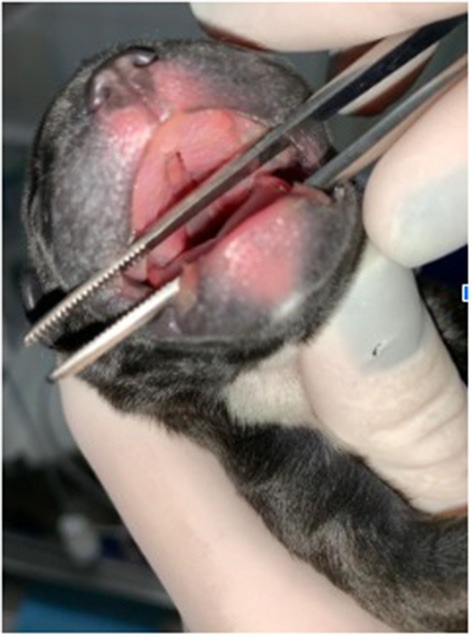
Cleft Palate in a French Bulldog neonate.

**Figure 2:**
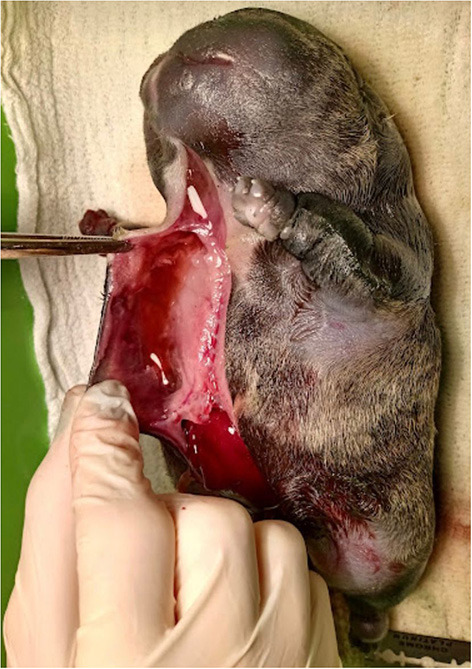
Anasarca in an English Bulldog neonate. Note the accumulation of fluid in the subcutaneous tissue, a remarkable characteristic of this type of malformation.

**Figure 3:**
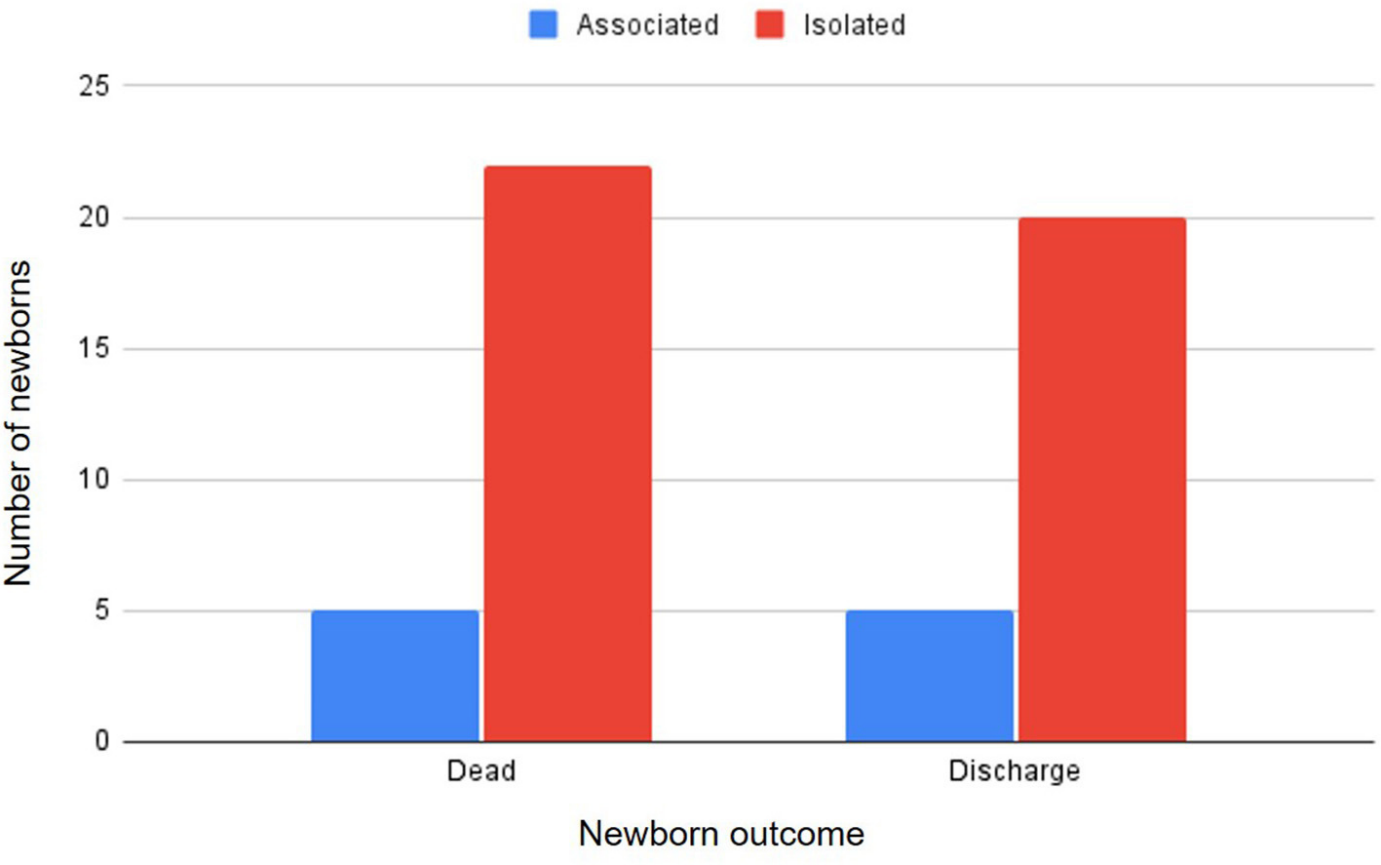
Type of congenital anomaly (isolated or associated) and neonatal outcome.

The total number of neonates delivered was 768 and 6.77% (52/768) of them were presented with CA. Overall, 23 different malformations were observed (Table 1). The most common malformations observed were palatoschisis (1.30%, Figure 1) and Anasarca (1.17%, Figure 2). Forty-two (80.7%) of the CA were single/isolated, whereas 10 (19.2%) were associated (Table 2). Of the 52 malformed cases, 27 (52%) died or were electively euthanized because of lethal malformations, with no difference of isolated and associated CA (Figure 3). Core variables such as birth weight and newborn sex could not be evaluated due to the missing data on medical records.

**Table 1:** Type of neonatal malformation and frequency by breed.

**Type of malformation**	**Frequency**	**Breed**
Palatoschisis	10/768 (1.30%)	French Bulldog, English Bulldog, mixed breed
Anasarca	9/768 (1.17%)	English Bulldog, mixed breed
Mitral dysplasia	6/768 (0.78%)	Pug
Omphalocele	4/768 (0.52%)	French Bulldog, English Bulldog,
Gastroschisis	4/768 (0.52%)	French Bulldog, English Bulldog, Shih Tzu, mixed breed
Renal dysplasia	4/768 (0.52%)	Pekingese
Hydrocephalus	3/768 (0.39%)	French Bulldog, Pug, German Spitz
Cheiloschisis	3/768 (0.39%)	English Bulldog, Shihtzu
Hypospadia	2/768 (0.26%)	Shih Tzu, French Bulldog
Swimming puppy syndrome	2/768 (0.26%)	Shih Tzu
Anencephaly	2/768 (0.26%)	Mixed breed
Atresia ani	2/768 (0.26%)	Shih Tzu
Rectourethral fistula	2/768 (0.26%)	Shih Tzu
Arthrogryposis	2/768 (0.26%)	MB
Eyelid aplasia	2/768 (0.26%)	MB
Macroglossia	2/768 (0.26%)	MB
Amelia	1/768 (0.13%)	French Bulldog
Lateralized anus	1/768 (0.13%)	French Bulldog
Urachus persistence	1/768 (0.13%)	French Bulldog
Spina bifida	1/768 (0.13%)	French Bulldog
Flexural deformity	1/768 (0.13%)	English Bulldog
Aplasia cutis	1/768 (0.13%)	Pinscher Miniature
Portosystemic shunt	1/768 (0.13%)	Pug

**Table 2:** Frequency, outcome, treatment, and description of associated congenital malformations by breed.

**Multiple congenital malformations**	**Breed**	**Frequency (%)**	**Newborn outcome**	**Treatment**
Amelia + hydrocephalus	French Bulldog	1/52 (1.92%)	Discharge	Clinical treatment for hydrocephalus
Omphalocele + palatoschisis	French Bulldog	1/52 (1.92%)	Dead	-
Palatoschisis + cheiloschisis	English Bulldog	1/52 (1.92%)	Discharge	Surgical correction
Anencephaly + palatoschisis + eyelid aplasia + macroglossia	Mixed breed	2/52 (3.84%)	Dead	-
Hydrocephalus + portosystemic shunt	Pug	1/52 (1.92%)	Discharge	Clinical treatment for both conditions
Atresia ani + hypospadias + rectourethral fistula	Shih Tzu	1/52 (1.92%)	Dead	-
Atresia ani + rectourethral fistula	Shih Tzu	1/52 (1.92%)	Dead	-
Artrogriposis + gastroschisis	MB	2/52 (3.84%)	Dead	-

Breeds having at least one malformed puppy were the English Bulldog (15/52), French Bulldog (12/52), ShihTzu (7/52), Pug (7/52), Pekingese (4/52), Miniature Pinscher (1/52), German Spitz (1/52) Lhasa Apso (1/52), and mixed breed (4/52). Table 3 summarizes the frequencies and percentages of congenital malformations stratified by breed.

**Table 3:** Number and (%) of litters, offspring, and malformed puppies by breed, attended in SORA (Service of Obstetrics and Animal Reproduction) from January 2017 to December 2021.

**Breed**	**N of litters (%)**	**N of offspring (%)**	**N of malformed puppies (%)**
French Bulldog	43/168 (25.59%)	193/768 (25.13%)	12/768 (1.56%)
English Bulldog	37/168 (22.02%)	187/768 (24.34%)	15/768 (1.95%)
Shih Tzu	18/168 (10.71%)	74/768 (9.63%)	7/768 (0.91%)
German Spitz	9/168 (5.35%)	27/768 (3.51%)	1/768 (0.13%)
Chow chow	8/168 (4.76%)	32/768 (4.16%)	0
Pinscher Miniature	8/168 (4.76%)	23/768 (2.99%)	1/768 (0.13%)
Pug	4/168 (2.38%)	18/768 (2.34%)	7/768 (0.91%)
American Bully	4/168 (2.38%)	27/768 (3.51%)	0
Lhasa Apso	3/168 (1.78%)	17/768 (2.21%)	1/768 (0.13%)
Border Collie	3/168 (1.78%)	21/768 (2.73%)	0
Maltese	2/168 (1.19%)	5/768 (0.65%)	0
Dachshund	2/168 (1.19%)	9/768 (1.17%)	0
Pekingese	2/168 (1.19%)	7/768 (0.91%)	4/768 (0.52%)
Rottweiller	2/168 (1.19%)	2/768 (0.26%)	0
Boxer	1/168 (0.59%)	9/768 (1.17%)	0
Poodle	1/168 (0.59%)	3/768 (0.39%)	1/768 (0.13%)
Blue Heeler	1/168 (0.59%)	6/768 (0.78%)	0
Labrador	1/168 (0.59%)	2/768 (0.26%)	0
Siberian Husky	1/168 (0.59%)	6/768 (0.78%)	0
Fox paulistinha	1/168 (0.59%)	4/768 (0.52%)	0
Yorkshire	1/168 (0.59%)	6/768 (0.78%)	0
Mixed breeds	16/168 (9.52%)	95/768 (4.55%)	4/768 (0.52%)

Brachycephalic breeds were 3.03 times more likely to have malformed neonates than other breeds; modern brachycephalic breeds are 5.07 times more likely to have malformed neonates than ancestral brachycephalics. Age seems to be a risk factor: mature bitches (above 7 years old) are 5.71 times more likely to have malformed puppies than young ones. On the other hand, when litter size was compared, no relation was found between the incidence of CA and the number of offspring in a litter (Table 4). Parity could not be evaluated as there were missing data on the medical records.

**Table 4:** Odds ratio, confidence interval, and significance according to independent variables of the dam.

**Independent variables of the dam**	**Description**	**Association with malformation**	**Odds ratio**	**Confidence interval**	***P value***
Breed	Brachycephalic Others purebred Mixed breed	Brachycephalic	3.03	95%	0.009
Age	Young (1-2y) Adult (3-6y) Mature (7-9)	> 7 years	5.71	95%	0.021
Mode of delivery	Elective c-section Therapeutic c-section Eutocic vaginal birth	Not related	-	-	-
Parity	Primiparous Multiparous	Missing data	-	-	-
Litter size	Small (1-4) Medium (4-6) Large (>7)	Not related	-	-	0.5996

Breeds with no congenital malformation reports in the present study were the American Bully, Blue Heeler, Border Collie, ChowChow, Boxer, Dachshund, Brazilian terrier, Siberian Husky, Labrador, Maltese, Poodle, Rottweiler, and Yorkshire. Regarding mode of delivery, 66.6% were elective c-sections (112/168), 19.64% (33/168) were eutocic vaginal deliveries, and 13.69% (23/168) were dystocic. There was no report of dystocia due to neonatal malformations; the bitches presented uterine inertia and were submitted to c-section.

## Discussion

According to the kennel club, the following breeds can be considered brachycephalic: Pug, French Bulldog, English Bulldog, Boston Terrier, Shih Tzu, Pekingese, Affenpinscher, Cavalier King Charles Spaniel, Lhasa Apso, and Griffon Bruxellois' (12). Many of these breeds were in our medical records. These dogs are characterized basically by short noses and wide heads. Associated with this conformation, problems such as brachycephalic airway syndrome (BAS), stenotic nares, elongated soft palate, narrowed trachea, visual problems, malocclusion, allergies, skin disorders, problems with heart performance, exercise intolerance, distichiasis, protrusion of the third eyelid gland, dystocia, neurological and spinal congenital malformations (7, 13, 14) are reported, including hemivertebrae and spina bifida, described in the present study.

Regarding the mode of delivery, there is a high incidence of elective cesarean sections (66.6%), due to the number of brachycephalic bitches attended at our veterinary hospital. Canine dystocia is a very common problem in clinical practice and can lead to the death of the dam and stillbirth (15). Risk factors for dystocia include breed, age of the dam, parity, litter size, and body size of the bitch. Fetal causes include fetal monster, anasarca, cephalopelvic disproportion, true fetal oversize or disproportion between fetal size and dam size, and fetal death (16). Knowing the risks of dystocia allows the best preparation for the intervention to be carried out at the right time and in the right way (15).

Besides the high rates of Cesarean section, the Pugs, Boston Terriers, and Bulldogs also seem to be most susceptible to neonatal mortality (14). The two most common causes of fetal depression associated with a cesarean section are hypoxia associated with dystocia and depression from medications given to the dam as part of the anesthetic protocol (16) thus, it is expected that breeds that are more likely to experience dystocia or that underwent elective c-sections have high rates of stillborn compared to breeds that can give birth without intercurrences. This information was confirmed by Cornelius et al. (15) who found out that puppies from litters in which a cesarean section was performed had 1.37 odds of being stillborn compared with litters that were delivered by vaginal route. Moreover, they reported that litters that experienced dystocia were 2.35 times more likely to have stillborn compared to the eutocic puppies. Within the last group, they notice that the management of dystocia also influences survival rates

Many studies have described the high incidence of dystocia in brachycephalic patients due to the physical conformation of the puppies (such as head size and chest width), the presence of oversized fetuses, and also fetal alterations such as anasarca and monstrosities, which justify elective c-section as the method of choice for these patients (13, 15, 17). In this study, 23 (13.69%) cases of dystocia ended up in c-sections. Of the 32 litters with malformed puppies, three were born from natural whelping, twenty-six from elective c-section, and three experienced dystocia (two due to uterine inertia and one caused by fetal malpresentation); none of them were caused by malformation.

Regarding the age of the dam, our data showed that mature bitches (over seven years of age) are more likely to deliver malformed puppies compared to young ones. To the authors' knowledge, this is the first study to show an association between aging and malformation in the canine species. In humans, on the other hand, there are robust data associating maternal age with the appearance of malformations: as age increases, it also increases the chance to develop birth defects (18). Whether or not there is a biological cause similar to that described in women must be further elucidated.

Many studies have been published reporting the frequency of specific malformations in dog populations (4, 7, 19, 20) but only a few presented an overview of the prevalence of these conditions in this species. Data obtained in the present research showed that 19.04% of the litters were affected and the incidence of malformations was 6.7%, rates notably higher than the 11.4 and 2.8% reported in the literature, respectively (1).

Among the thirty-two litters that presented malformations, 87.5% were brachycephalic: English Bulldog (10/32), French Bulldog (9/32), ShihTzu (5/32), Pug (2/32), Pekingese (2/32) and Lhasa apso (2/32); moreover, 19 of the 21 types of malformations were recorded in brachycephalic dogs.

Twenty-three types of malformations were observed, the most common being cleft palate (1.3%) and anasarca (1.17%). A large study conducted in Brazil that included 27 types of malformations and 23 breeds showed that 24.7% of litters had some type of malformation, with 6.7% of the neonates affected. The most common condition was cleft palate (2.8%) and hydrocephalus (1.5%) (3), which are in accordance with our findings. A study conducted in Australia reported that 11.4% of litters were born with congenital defects, which represented 2.8% of the neonates (1).

Orofacial clefts are fissures of oral or facial structures that occur during embryonic development. Several studies suggested that the phenotypic manifestation of the cleft in dogs is associated with skull type (4). In these species, the alteration is clinically relevant because of the associated morbidity and high mortality rate (4). The mastiff/terrier genetic group includes most brachycephalic breeds such as Boston Terrier, English Bulldog, and French Bulldog and they seemed to be more predisposed to orofacial clefts (4, 21). Interestingly, the morphology of the cleft appears to be different according to the type of the skull: compared to other breeds, brachycephalic dogs seem to be more prone to cleft lip or cleft lip associated with cleft palate than to cleft palate (21).

A study focused only on orofacial clefts found a phenotypic distribution as follows: 26% of Cleft Lip (CL), 59% of Cleft Palate (CP), and 15% of Cleft Palate and Lip (CPL) (4). Our study shows a similar distribution: of the 12 puppies that had orofacial clefts, 2 presented only CL (16.6%), 9 presented only CP (75%) and just one presented CPL (8.3%). Although our frequencies are different, the authors found out that Brachycephalic breeds were at increased odds of orofacial clefts of any type (4). Indeed, in our study, of 12 puppies with orofacial clefts, only two were Mixed breed and had other associated malformations. All the others were brachycephalic: two Shih Tzu puppies (CL), seven French Bulldogs (CP), and One English Bulldog (CPL). The frequency of orofacial clefts observed in other studies varied according to phenotype (CL vs. CP vs. CLP) and reinforces the hypothesis that the CP phenotype in dogs is more common compared to CL and CLP (4, 21). However, results also showed that this pattern may only apply to certain breeds (e.g., Labrador Retriever, Pembroke Welsh Corgi, and French Bulldog) and not to others (e.g., Boston Terrier, Cavalier King Charles Spaniel, English Bulldog). Accordingly, our results showed that in French Bulldogs, CP is the most common orofacial cleft, while in English Bulldogs, CLP was the most prevalent. Specifically, the odds of CP, CL, and CLP were consistently and significantly higher in the brachycephalic group compared to the reference skull type group (i.e., mesaticephalic) (4). Even though our sample for CPL was too small, we observed a similar distribution and tendency. The treatment of oral clefts is mostly surgical (21) and so our patients were referred to general surgery service and we had no further information about their outcome.

Some authors pointed out a strong influence of the genetic factor on the pathogenesis of orofacial clefts, (4, 21) however, one may not exclude the influence of environmental factors such as geographic region (considering exposure to pathogens) and rearing method (nutritional factors, folic acid deficiency, trauma, and drug use) (4).

The comparison of our results with other Brazilian studies (especially those conducted in the same microregion) is essential due to the geographic issue. Studies conducted in other countries may take into account cultural differences in breeding (such as nutritional management, and infectious diseases) that may affect the incidence of malformation. There was no record of trauma, contact with teratogens, or marked nutritional characteristics in any of the cases of cleft palate observed in this study, but the high incidence in brachycephalic patients (8/10) once again highlights the importance to consider genetic traits.

Anasarca was the second most frequent malformation (9/52) recorded in the present study, with a high incidence in English Bulldogs (8/9). This condition, also called congenital edema or hydrops fetalis, is known to be a heritable recessive trait that involves generalized subcutaneous edema with or without visceral effusions (2, 10, 22).

The cause is unknown, but some studies point out associated cardiac malformations, infectious causes, and, again, the genetic factor (2, 22). It is important to notice that five of the nine puppies that presented anasarca were from different litters, but originated from the same commercial breeder, which once again denotes inbreeding.

The condition often causes dystocia due to a typically enlarged fetus and is usually associated with a high neonatal mortality rate and a greatly increased rate of cesarean sections. The English Bulldog, the Pug, the Boston Terrier, and the French Bulldog all have increased incidence of fetal anasarca and this condition may involve the whole or part of the litter (2, 10, 22). In this study, two of the English Bulldog litters presented more than one anasarca. Although it is associated with high neonatal mortality, there are scarce reports of successful treatment of anasarca's fetuses (22). Unfortunately, all anasarca in this study have some lethal condition (such as pulmonary hypoplasia or bilateral hydronephrosis), so they were euthanized immediately after birth. It is important to point out that puppies who go through treatment and survive, must be neutered.

The present study also described a high frequency of mitral dysplasia (0.78%). However, it is important to consider that the six affected Pug puppies were from the same litter whose mother also presented the condition, suggesting a possible hereditary condition. The same malformation was described in a similar study in a French Bulldog puppy, but the reported incidence was 0.12% (1). Among the cardiac malformations, Mitral dysplasia is one of the most common and has a high incidence in Terrier dogs, with low evidence in Bulldogs (19, 23) and no data available in Pugs. The exact prevalence of heart disease is difficult to estimate because not all patients have heart murmurs.

It is well known that during pregnancy, there are many adaptations in cardiac function, probably due to hemodynamic changes. Blanco et al. (24) described the systolic cardiac function and peripheral circulation changes in pregnant bitches. In this species, maternal cardiac adaptation during gestation plays a major role in uterine perfusion to support fetal development. Comparing echocardiographic parameters in normal and abnormal pregnancies, Blanco et al. (25) reported differences in left ventricular dimension in diastole (LVD), HR, FS, Systolic Volumes (SV), and CO between the two groups. This may point to a possible cardiac maladaptation to pregnancy in bitches with the abnormal gestational course. For this reason, it would be interesting to carry out preventive cardiological examinations in predisposed breeds (Boxer, Newfoundland, French Bulldog, English Bulldog, German Shepherd, Golden Retriever, and Labrador Retriever) before the animals are mated (19).

Melandri et al. (26) described an increase of functional and diastolic parameters and the decrease of systolic parameters as pregnancy progresses in healthy Great Dane bitches, a breed particularly predisposed to dilated cardiomyopathy and Subaortic Stenosis (23, 26). This finding reinforces the importance of echocardiographic examination as part of prenatal follow-up in breeds predisposed to cardiac diseases. The authors also consider Breed as a source of variation in echocardiographic values, therefore the cardiac performance during pregnancy may also be susceptible to a breed-related variation. Once again, pugs are not predisposed to mitral dysplasia and the bitch had no clinical signs, so this result was an incidental finding.

Hydrocephalus was reported as the second most common malformation in a recent study (3), but the rate of this condition in our study was notably lower. This difference might be attributed to the absence of a diagnosis since part of the data was retrieved from medical records. Dogs and cats with congenital hydrocephalus may have signs from birth such as a large, dome-shaped head, persistent fontanelles, and bilateral ventrolateral strabismus that may be the result of either orbital skull malformation or vestibular dysfunction, however, more commonly signs become apparent in the first few months of life (27). Moreover, hydrocephalic puppies often show a retarded growth, so comparison with littermates can be very helpful (28). Unfortunately, the progression of symptoms is variable, so affected puppies may not show clear neurological signs, especially when they are very young (28). Therefore, those puppies who did not present apparent malformations or clinical signs at birth may not have been registered as malformed in the medical records and so the incidence rate may be underestimated.

Reduced cranial capacity impairing cerebral compliance and malformations of the craniovertebral junction (atlantoaxial instability, occipital-atlantoaxial overlap syndrome, and “Chiari-like malformation”) are the most common causes of impaired CSF flow and communicating hydrocephalus in a high number of brachycephalic breeds (28).

Congenital hydrocephalus is frequently diagnosed in toy breed dogs including Pomeranians but is also overrepresented by the brachycephalic group (Boston terrier, English Bulldog, Maltese, Pug, Pekingese, and Chihuahua) (27, 28). The latter is the most commonly affected breed, which might indicate that genetics play a role in the pathogenesis of hydrocephalus in this breed (28). Once again, our sample was too small to identify a breed predisposition, but the affected puppies belonged to breeds that seem to be more prone to present that malformation according to the literature. Medical therapy involves decreasing the production of CSF with diuretics, omeprazole, and glucocorticoids associated with antiepileptic drugs, and the most common surgical treatment is the placement of a ventriculoperitoneal shunt (27). When surgical treatment is indicated, patients are referred to the General Surgery Service.

Congenital vertebral malformations are so common findings on diagnostic imaging of the vertebral column in “screw-tailed” brachycephalic patients that 51% of the dogs evaluated in a study had evidence of one or more lumbosacral congenital vertebral malformations. These alterations had a high prevalence even in neurologically normal French Bulldogs, English Bulldogs, and Pugs (6).

In our study, only one French bulldog puppy was diagnosed with spina bifida (0, 13%), and one female that gave birth to one CP puppy already had a previous litter with one hemivertebrae puppy.

Although these changes are diagnosed in animals without clinical signs, Vertebral body malformations are suggested to change spinal biomechanics that can lead to premature degeneration of adjacent intervertebral disk (6). In one study, French Bulldogs showed 0.89 times the odds of being diagnosed with at least a disease compared to other breeds (29).

Of the 32 affected litters, 28 were purebred, from which 21 were from commercial breeding kennels and two of these dams had already records of previous litters with malformations. Breeding for a commercial purpose is usually controlled, to modify specific physical or behavioral traits, or aim at improving health and genetic diversity; however, multiple generations of inbreeding to fix a specific trait can result in offspring with a lack of genetic diversity (30). In addition, dogs that are not wanted for breeding purposes are commonly neutered, so the reproductive population is much smaller than the census population, decreasing genetic variability (30). Genetic effects of inbreeding can be attributed to the fact that the inbred individual may carry two copies of a gene (31), including the deleterious alleles. For this reason, inbreeding influences the incidence of some inherited diseases (31).

Considering only the gross defects previously presented in our study, brachycephalic breeds are 3.03 times more likely to have congenital malformations than other skull types, especially if they have extreme traits.

Many modern brachycephalic breeds are characterized by a shortening of the muzzle bones of the skull without an equivalent reduction in the volume of the associated nasopharyngeal soft tissues that predisposes to clinical upper respiratory tract disorders (32) known as brachycephalic airway syndrome (BAS). Studies showed that the frequency of BAS was higher in modern compared to ancient breeds. This confirmed the findings that boxers are not as susceptible to BAS as many other brachycephalic breeds belonging to the same phylogenetic cluster (33).

Comparison between extreme brachycephalic breeds (English Bulldog, French Bulldog, ShihTzu, and Pugs), known as modern breeds, with ancestral brachycephalic breeds showed that the odds ratio rises to 5.07. This finding highlights the importance to re-evaluate animal selection and breeding management.

The study of Njikam et al. (33) on BAS reported differences between ancestral and modern brachycephalic breeds, which might indicate that some traits are not derived from the same ancestral characteristics. Although one might assume that dog breeds constitute homogenous entities, the popularity of some breeds may have created isolated subsets of dogs within some breeds (33).

A study that estimated the inbreeding coefficient, found that brachycephalic breeds (including those previously cited) had a high degree of homozygosity (30). They also noticed that nearly all of the dogs with high inbreeding coefficients belonged to brachycephalic breeds, which are likely to have reduced lifespans due to specific pathologies imposed by their skull morphology (30).

The same pressure that selects these extreme phenotypic characteristics and brings the intrinsic problems of the conformation (such as respiratory, locomotor, and reproductive difficulties) also selects for serious congenital malformations that worsen the quality of life and increase neonatal mortality. Even malformations that have treatment, cause a lot of suffering for both the puppy, who may need careful and stressful handling, surgeries, and medication, and for the owner, who will spend a lot of money and time for the treatment, not to mention the potential suffering for the bitch in cases of dystocia of fetal origin.

To improve the quality of life for dogs, breeding purebred dogs must be carefully thought out. Due to their extreme characteristics, many brachycephalic patients only reproduce through artificial insemination and cesarean sections. One of the ways to select healthier animals is not to cross animals that only reproduce with the use of biotechniques (34). Breeding standards should be revised and not be left open to interpretation allowing the perpetuation of traits with a negative impact on the health and welfare of the dogs (34).

Limitations inherent to retrospective studies and present in this one includes data loss and unknown confounding factors, which did not allow us to correlate the incidence of the malformations and the sex of the neonates, as well as to calculate the mortality rate. Unfortunately, many puppies born in the sector without apparent malformations, died after being discharged from the hospital and breeders did not always get in touch to report the death or to request a necropsy study.

## Conclusion

In summary, the incidence of malformations in brachycephalic breeds is notably higher when compared to other morphological types, especially when considered the ones presenting with extreme traits, which can be attributed, most likely, to the high level of inbreeding. Anasarca and orofacial clefts are the most common malformations, which prompted the need for additional studies about their pathophysiology and treatment. Moreover, investigations focusing on the determination of the genetic role in malformations are of pivotal importance for the veterinarians to be able to accurately instruct breeders in the selection of animals to be acquired and reproduced, as well as to choose the appropriate prenatal care, delivery, and neonatal approach.

## Data availability statement

The raw data supporting the conclusions of this article will be made available by the authors, without undue reservation.

## Ethics statement

The animal study was reviewed and approved by Ethical Committee of the São Paulo State University. Written informed consent was obtained from the owners for the participation of their animals in this study.

## Author contributions

All authors listed have made a substantial, direct, and intellectual contribution to the work and approved it for publication.

## Conflict of interest

The authors declare that the research was conducted in the absence of any commercial or financial relationships that could be construed as a potential conflict of interest.

## Publisher's note

All claims expressed in this article are solely those of the authors and do not necessarily represent those of their affiliated organizations, or those of the publisher, the editors and the reviewers. Any product that may be evaluated in this article, or claim that may be made by its manufacturer, is not guaranteed or endorsed by the publisher.
